# The prevalence of grandparental childcare in Europe: a research update

**DOI:** 10.1007/s10433-023-00785-8

**Published:** 2023-09-25

**Authors:** Francesca Zanasi, Bruno Arpino, Valeria Bordone, Karsten Hank

**Affiliations:** 1 University of Bologna, Bologna, Italy; 2 University of Padua, Padua, Italy; 3 University of Vienna, Vienna, Austria; 4 University of Cologne, Albertus Magnus Platz, 50923 Cologne, Germany

**Keywords:** Grandparents, Grandparental childcare, SHARE, Europe

## Abstract

We investigate (a) how the proportion of European grandparents providing childcare changed over a period of 15 years, (b) how these proportions differ by gender and education, and (c) how countries not covered in earlier analyses fit into previously identified regional patterns of grandparental childcare in Europe. Using data from Waves 1, 2, and 8 of the Survey of Health, Ageing and Retirement in Europe (SHARE), our descriptive analysis provides estimates of the prevalence and intensity of grandparental childcare in 26 European countries as well as of the changes therein over time and across socio-demographically defined groups. Overall, the prevalence and intensity of grandparental childcare in Europe has remained fairly stable over time, with minor increases. Proportions of grandparents providing any childcare strongly vary, however, across countries (from 24 to 60%). Grandmothers are generally more likely to provide childcare than grandfathers, while differences based on educational levels are less clear-cut. Central and southeastern Europe, representing the bulk of the ‘new’ countries in the analysis, exhibit patterns of grandparental childcare closely resembling those observed in Mediterranean countries. Our analysis revealed an overall stability over time rather than change in grandparents’ provision of childcare in Europe, with substantial variations across welfare state regimes and within countries when accounting for grandparents’ gender and educational levels. Including countries that had previously been excluded from other studies challenges the ‘narrative’ that has emerged around a negative macrolevel association between the provision of extensive and intensive grandparental childcare.

**Supplementary Information:**

The online version contains supplementary material available at 10.1007/s10433-023-00785-8.

## Introduction

Whereas the role of a grandparent encompasses various dimensions (such as expectations, meaning, and satisfaction; Thiele and Whelan 2006), empirical studies have mainly focused on active grandparenting, specifically on grandparental childcare (see Bordone et al. 2023). These studies have investigated the consequences of grandparental childcare for a variety of outcomes across generations in the family—such as grandparents’ health (e.g., Danielsbacka et al. 2022) or grandchildren’s cognitive outcomes (e.g., Del Boca et al. 2018)—and identified that family members’ resources and needs are important determinants in the provision of grandparental childcare (e.g., Hank et al. 2018). Moreover, cross-national comparisons have pointed to substantial variations in the prevalence and intensity of grandparental childcare in different welfare state contexts (Bordone et al. 2023).

Comparative research on grandparental childcare in Europe conducted over the past 15 years or so has predominantly used data from the Survey of Health, Ageing and Retirement in Europe (SHARE; see Bordone et al. 2017; Di Gessa et al. 2016; Hank and Buber 2009; Igel and Szydlik 2011, for example), building on baseline interviews mainly collected in 2004–05 (Wave 1) and, for a few countries that joined SHARE in Wave 2, in 2006–07. Since then, however, the provision of public day care for children has become more readily available, and older workers’ (especially women’s) participation in the labor force has increased in many countries, which may have affected the prevalence and intensity of grandparents’ provision of childcare. Moreover, SHARE’s baseline waves covered 13 European countries (plus Israel), whereas its most recent Wave 8 (conducted in 2019–20) collected data in twice as many countries, also comprehensively covering Central and Eastern Europe.

Complementing information from SHARE’s baseline data with data from Wave 8, our descriptive study therefore aims to answer three questions: *First*, (how) has the share of grandparents providing childcare changed over a period of 15 years across European countries? *Second*, (how) does this share differ according to relevant sociodemographic characteristics? As recent research has indicated, distinct patterns in the involvement of grandparents with their grandchildren exist depending on the grandparents’ gender and level of education (e.g., Craig et al. 2019; Di Gessa et al. 2022; Zanasi & Sieben 2022); therefore, we specifically focus on these two characteristics. *Third*, how do ‘new’ SHARE countries that were not examined in earlier studies fit into previously identified patterns of grandparental childcare (along the lines of a ‘familialism by default’ to ‘defamilialisation’ continuum; Bordone et al. 2017)? We thereby aim to provide an up-to-date empirical basis for further academic and policy debates about the role of grandparents as childcare providers in the family.

## Grandparental childcare from a comparative perspective

In the long-lasting debate over the relationship between family solidarity and the role of welfare, some authors have claimed that generous welfare states crowd out family solidarity, while others have argued that welfare services may instead stimulate and thus crowd in intergenerational support within the family (Daatland and Lowenstein 2005; Künemund and Rein 1999). More recently, research drawing on the concept of mixed responsibilities or *specialization* (Brandt 2013; Brandt et al. 2009; Igel et al. 2009) has shown that family and state provisions interact (Brandt et al. 2009; Motel-Klingebiel et al. 2005). In universalistic northern welfare states, family members economically and socially support each other more often (crowding-in) but with lower intensity (crowding-out) than in southern European countries, where there is a polarization between high-intensity support that is exchanged within the family and no support at all (Albertini et al. 2007). This divide reflects an association between more generous welfare contexts, where time-consuming and specialized tasks are carried out by public services, and family members taking on support tasks voluntarily rather than by obligation to support family members (Brandt 2013).

Grandparental childcare, as a form of intergenerational exchange, also follows this pattern. Among the roughly half of European grandparents involved in childcare (Hank and Buber 2009), southern Europeans are less likely to engage in grandparental childcare, but when they do, it is on a more intense basis (e.g., weekly or daily) compared to the type of childcare provided by their northern European counterparts (Hank and Buber 2009; Igel and Szydlik 2011). Compositional factors, such as demographic and socioeconomic characteristics of both parents and grandparents, partly contribute to this heterogeneity. For example, parents are more likely to be married, older, and to have only one child in Italy, Greece, and Spain than in Northern or Central Europe. Furthermore, grandparents are more likely to have a lower level of education and not be engaged in paid work in Mediterranean countries than in other European countries (e.g., Glaser et al. 2013; Hank and Buber 2009). Additionally, individuals tend to become grandparents at older ages in Southern Europe (Skopek 2021; Zanasi and Sieben 2020). However, such variation explains relatively little of the wider cross-national variation in intensive grandparental childcare (Di Gessa et al. 2016) that rather seems attributable to the considerable heterogeneity in macrolevel factors, such as the generosity of welfare services, labor market structure, and cultural norms. Among the studies on the relation between welfare policies and grandparental childcare (Aassve et al. 2012; Herlofson and Hagestad 2012; Igel and Szydlik 2011; Price et al. 2018), Bordone et al. (2017) identified three models of grandparental childcare by applying Saraceno and Keck’s (2010) threefold conceptualization of family policies.

In Mediterranean countries but also in Poland, *familialism by default*, or unsupported familialism, prevails: on the one hand, there are no or little publicly provided alternatives to family care, which forces family members to step in; on the other hand, as women are mainly economically inactive, there is little need for grandparents to support childcare. Whenever women are employed, however, the need for childcare is high, requiring that grandparents who do care for their grandchildren engage in childcare mainly daily to substitute for (scarce) public childcare. Moreover, this situation is reinforced by the low availability of part-time jobs in these countries (Bordone et al. 2017; Di Gessa et al. 2016). According to Saraceno and Keck’s (2010) categorization, this situation also prevails in Bulgaria, Latvia, and Slovakia, but no comparative study has investigated grandparental childcare in these countries thus far.

Nordic countries and France are characterized by *defamilialisation*, since in those places public or publicly financed and regulated services relieve families from their duties, as well as by *supported familialism,* that is, policies that financially support families in maintaining their caring role. Allied with the fact that part-time jobs are vastly available in those countries, these policies lower the need for grandparental childcare, even when young mothers are extensively employed. Grandparents can thus engage in grandchild care when they want to (Igel et al. 2009; Igel and Szydlik 2011) or in case of emergencies.

An intermediate and more heterogeneous model characterizes most of the Western European countries (Austria, Belgium, Germany, and the Netherlands) and the Czech Republic, where levels of policy support and the extent to which women participate in the (part-time) labor market range between the levels shown in the other two models. In these countries, grandparents are largely involved in grandchild care but usually on a weekly basis, as a complement of public services. In this scenario, too, the categorization by Saraceno and Keck (2010) included additional countries that could not be tested by Bordone et al. (2017): Estonia and Hungary, showing levels of supported familialism close to the Czech Republic; and Luxemburg, more similar to the Netherlands and Austria in terms of familialism by default.

Since the first two waves of SHARE (on which most of the studies cited above were based), in several European countries, public day care for children has become more readily available, and older workers’ participation in the labor force has increased (especially women’s), which may have affected the prevalence and intensity of grandparents’ provision of childcare. A recent Italian study still found patterns of stability rather than change in this respect (Pasqualini et al. 2021). Our study expands this research in a comparative perspective, updating the statistics on grandparental childcare for those countries that have already been treated in the literature and including ‘new’ countries by exploiting data from SHARE that now allow us to more comprehensively analyze Central and Eastern Europe. We thus provide background knowledge to scholars and policymakers.

## Differences in childcare provision according to gender and education

In this study, grandparental childcare is studied separately according to the gender and educational level of the grandparents. It is well established that a gendered division of labor exists within couples, first and foremost as far as care provision is concerned. This division persists in later life, with grandmothers providing childcare to a greater extent than grandfathers (Craig and Jenkins 2016; Hank and Buber 2009; Leopold and Skopek 2014). Although grandparenthood is highly valued by men as an opportunity to make up for the time lost with their own children (Airey et al. 2020; Mann 2007), grandfathers have thus often been excluded from research focused on grandparents. Recently, Coall et al. (2016) talked about “new grandfathers” to refer to men who actively engage as carers rather than simply as helpers for the female partner. Empirical evidence thus far has in some cases highlighted grandfathers’ contributions as confined to leisure activities (Dunifon et al. 2018; Horsfall and Dempsey 2015) or led to the conclusion that there was no difference across genders (Di Gessa et al. 2020). Given how heterogeneous gender roles are within families and the differences in the availability of grandmothers and grandfathers (e.g., due to employment and retirement patterns) in different European countries, we expect to find differences in grandparental childcare provision between grandmothers and grandfathers in the countries we investigate.

Education can be considered a proxy for cultural capital and childrearing style but also for the resources that grandparents can devote to their grandchildren (both in terms of income and access to health). Several studies have shown a positive educational gradient in the probability of grandparents providing childcare in terms of occurrence (e.g., Craig and Jenkins 2016; Dunifon et al. 2018; Igel and Szydlik 2011; Zamberletti et al. 2018). When considering intensity, however, higher education acts as a protective factor against an intensive commitment (Di Gessa et al. 2016). The two mechanisms at play have been discussed in previous studies (e.g., Arpino et al. 2018). Education is indeed selective with respect to grandparental childcare serving as a resource; grandparents with higher levels of education are better integrated into the family network, but grandparents with higher levels of education also prefer to participate in the labor market and other social activities, which reduces how intensely they provide childcare. Given that grandparents with higher (lower) education are likely to also have children with higher (lower) education, greater demands for grandparent childcare when grandparent are more highly educated may also result from aspects linked to the greater educational attainments of their own children, which positively associate with the values that promote women in the workforce. Along these lines, Zanasi et al. (2022) found that grandmothers who used to work are more likely to provide grandparental childcare, especially when parents work, compared to grandmothers who never worked.

## Data and method

*Data & sample.* Our analysis builds on the Survey of Health, Ageing and Retirement in Europe (SHARE; Börsch-Supan et al. 2013). More specifically, we use baseline interviews from Wave 1 (2004–05) for most of the countries and Wave 2 (2006–07) for those countries that joined SHARE later, and we include refresher samples (i.e., respondents from countries already included in Wave 1 who were first interviewed during Wave 2). We combine these interviews with all those from the most recent, regular (that is, pre-COVID-19) Wave 8, collected in 2019–20 (see Börsch-Supan 2022a, b, d). Note that Wave 8’s fieldwork was interrupted in most countries at the onset of the COVID-19 pandemic, which means that all data refer to the pre-pandemic period. This detail is important, as grandchild care provision has been affected by the pandemic (Di Gessa et al. 2023).

Baseline interviews were conducted in 13 European countries (excluding Israel), namely, Austria, Belgium, the Czech Republic, Denmark, France, Germany, Greece, Italy, the Netherlands, Poland, Spain, Sweden, and Switzerland. By Wave 8, 13 additional countries were added to the survey, namely, Bulgaria, Croatia, Cyprus, Estonia, Finland, Hungary, Latvia, Lithuania, Luxembourg, Malta, Romania, Slovakia, and Slovenia. Thus, a total of 26 European countries contributed data to SHARE’s most recent wave. Both our analytic samples (baseline and Wave 8) consist of respondents aged 50 years or older who reported having at least one grandchild at the time of the interview, amounting to 24,596 individuals in the ‘baseline’ sample and 33,625 individuals in Wave 8.

Importantly, respondents in Wave 8 predominantly contributed follow-up interviews. As the fieldwork had to be stopped in March 2020 due to the outbreak of the COVID-19 pandemic, in several countries the drawn refreshment samples could not be fielded at all or in part (for details, see Bergmann and Börsch-Supan 2021); therefore, respondents tended to be older than those in the baseline sample. Calibrated weights provided by SHARE partially addressed the problem (see Additional file 1:﻿ Figure S1). In addition to adjusting for age (unadjusted estimates are reported in Additional file 1: Table S4), we performed further robustness checks to ensure that our comparisons of estimates across time were not affected by differences in the samples’ age distributions. We estimated predicted probabilities using SHARE’s Wave 6 (Börsch-Supan 2022c), which was conducted in 2015 and includes a substantial refreshment sample, and we restricted both our analytic samples to individuals aged 60 or older. These checks (see Additional file 1: Tables S1 & S4) provided no indication that differences in the age distribution across the different waves matter for the results presented below.

*Variables.* Respondents were asked whether, during the last twelve months, they had looked after any grandchild(ren) without the presence of the grandchild(ren)’s parents, and if so, how often. From the answers provided to these questions, we derived two binary indicators: the first one took a value of 1 if the responding grandparent provided ‘any care’ at all during the twelve months preceding the interview and 0 otherwise. The second indicator distinguished grandparental childcare intensity, namely, it distinguished between grandparents who looked after grandchildren on an ‘at least weekly’ basis from those who did so less regularly (or not at all). Moreover, we accounted for respondents’ country of residence and age (in all models), eventually performing separate analyses by gender (male vs. female; Fig. 2 & ﻿ Additional file 1:﻿ Table S2) and education (primary/secondary vs. tertiary; Fig. 3 & Additional file 1: Table S3), respectively.

*Method.* Logistic regression models were employed to estimate the proportion of grandparents providing ‘any’ (vs. none) or ‘at least weekly’ (vs. less) childcare (for a similar approach see e.g. Hank and Buber 2009). First, we estimated models that regressed any and weekly care on wave, country dummies, and age and the tree-way interaction ‘wave, country, age’. Then, to test within-country changes in grandchild care provision across gender and education groups, we ran two additional sets of models while adding two interaction terms: ‘wave, country, age, gender’ and ‘wave, country, age, education’. Standard errors were clustered at the individual level to account for repeated observations (20% of the sample answered in both waves). Calibrated weights were applied in all models. The results are presented graphically in terms of predicted probabilities.

## Results

**Fig. 1:**
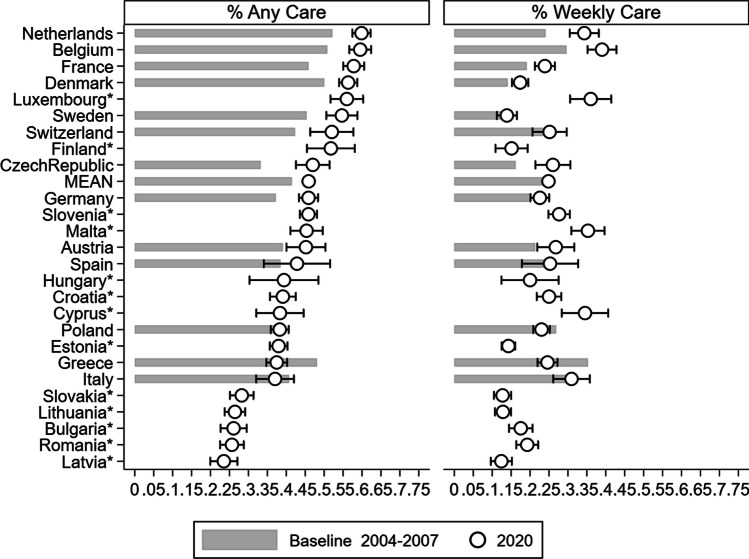
Percentage of grandparents providing any (left panel) and at least weekly (right panel) childcare: Comparison between baseline (2004–07) and Wave 8 (2019–20) *Note*: Predicted probabilities from age-adjusted logistic regression models. Calibrated weights applied. *95% confidence intervals* shown for* Wave 8 (2019–20).* Confidence intervals for baseline (2004–2007) and tests of differences between* Wave* 8 and baseline wave are available in Additional file 1: Table S1.* Sorted by percentage of grandparents providing any care in 2019–20.* Asterisks:* ‘new’ (Wave 8, 2020) countries.* *Numerical values are available in the Supplementary Materials*

Figure 1 displays the adjusted predicted probabilities of ‘any’ and ‘at least weekly’ (that is, regular) grandparental childcare by country and wave (see Additional file 1: Table S1 for the corresponding numerical values and for the test of differences between Wave 8 and the baseline wave). The probability of providing *any* grandchild care in 2019–20 varies between 24% in Latvia (closely followed by Romania, Bulgaria, and Lithuania) and 60% in Belgium and the Netherlands (closely followed by France and Denmark), with an average of 46%. When comparing the 13 countries that participated in both the 2004–07 and 2019–20 waves, an overall picture of stability over time emerges; if anything, most changes appear within a range of 10 percentage points (pp), usually denoting a modest increase in the proportion of grandparents providing any childcare—irrespective of a country’s baseline level. Substantial increases are seen in only a few countries, such as the Czech Republic (from 33 to 47%) and France (from 46 to 58%). An exception to this general pattern is Greece, where we observe a considerable drop (from 48 to 38%). The Spearman correlation between the estimated prevalence of grandparental childcare at baseline and follow-up is 0.60, meaning that the ranking of countries between the two waves is slightly altered.

The prevalence of *regular* grandchild care in 2019–20 varies between 13–17% in the Baltic (Estonia, Latvia, and Lithuania) and Nordic countries on the one hand and 39% in Belgium (closely followed by several smaller countries: Cyprus, Luxembourg, and Malta) on the other hand; the average here is 25%. We observe, again, a high level of stability over time: only three countries show increases of more than 5 percentage points (Belgium, the Czech Republic, and the Netherlands), whereas Greece is once more the sole outlier, exhibiting a substantial decrease (from 35 to 25%). The case of Greece stands out: if one also considers Wave 6 (see Additional file 1: Table S1), the drop in any childcare does happen after Wave 6, which could lead to consideration related to the sample’s composition (e.g., age distribution shifted toward older individuals). However, additional checks only on grandparents who are 60 years and older (see Additional file 1: Table S4) confirmed this drop. The drop in regular grandchild care, instead, is more gradual (5 percentage points from the baseline to Wave 6 and 5 percentage points from Wave 6 to Wave 8). Given this outlier, the Spearman correlation between the estimated prevalence of regular childcare in 2004–07 and 2019–20 is 0.54.

The ‘new’ countries that did not already contribute to SHARE’s baseline wave take various positions in the overall ranking of countries: the prevalence of grandparental childcare in 2019–20 in Finland and Luxembourg, for example, is similar to that in other northern and continental European countries (such as France or Sweden), whereas the patterns in central and southeastern Europe closely resemble those observed in Mediterranean countries such as Italy or Greece.

**Fig. 2:**
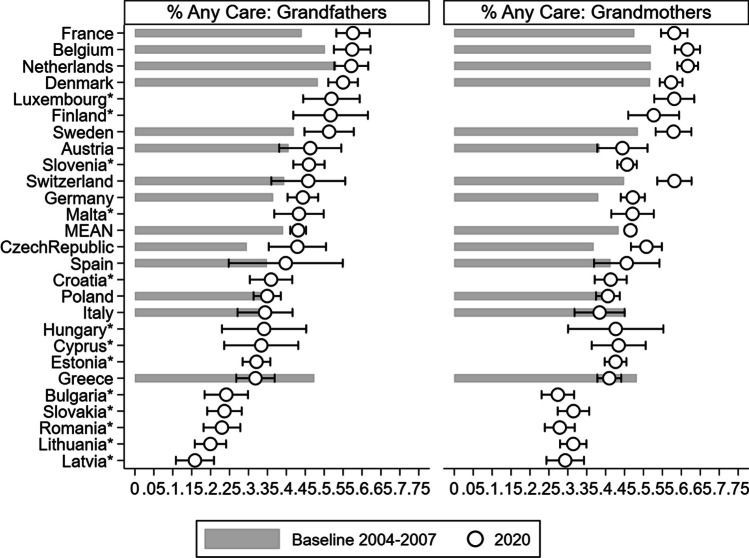
Percentage of grandparents providing any childcare: Comparison between baseline (2004–07) and Wave 8 (2019–20), by gender *Note: Predicted probabilities from age-adjusted logistic regression models. Interaction term between country and gender included. Calibrated weights applied.* 95% confidence intervals shown for* Wave 8 (2019–*20). Confidence intervals for baseline (2004–2007) and tests of differences between* Wave* 8 and baseline wave are available in Additional file 1: Table S2.* Sorted by percentage of grandfathers providing any care in 2019–20. Asterisks: ‘new’ (Wave 8, 2019–20) countries.* *Numerical values are available in the Supplementary Materials*

Figure 2 shows the predicted probabilities of any grandparental childcare by country, wave, and *gender* (see Additional file 1: Table S2 for results referring both to any and to regular childcare by gender). For the general pattern, the prevalence of grandparental childcare is fairly stable over time for both grandfathers and grandmothers. Moreover, the ranking of countries in terms of grandparental childcare provision is similar for both genders and to the general pattern shown in Fig. 1. However, grandmothers exhibit an overall higher probability of providing childcare in most countries in both periods (47% vs. 43% for grandfathers on average in 2019–20), albeit limited in scope, that is, hardly exceeding 10 percentage points. In Wave 8, when excluding Switzerland (12 pp), a wider gender gap in ‘any’ grandparental childcare occurs in the ‘new’ countries, that is, Estonia (11 pp), Lithuania (11 pp), and Latvia (13 pp); only Switzerland (16 pp) and Latvia (11 pp) exceed this level for regular grandchild care. Finally, in countries that experienced a substantial change in the proportion of grandparents providing childcare, both genders contributed to the observed increase (France) or decrease (Greece).

**Fig. 3:**
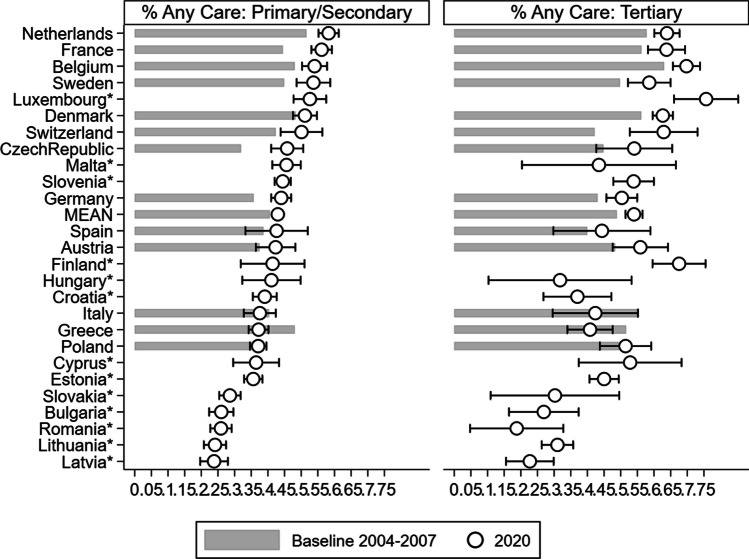
Percentage of grandparents providing any childcare: Comparison between baseline (2004–2007) and Wave 8 (2019–20), by educational level *Note: Predicted probabilities from age-adjusted logistic regression models. Interaction term between country and educational level included. Calibrated weights applied.* Calibrated weights applied. 95% confidence intervals shown for* Wave 8 (2019–*20). Confidence intervals for baseline (2004–2007) and tests of differences between* Wave* 8 and baseline wave are available in Additional file 1: ﻿Table S3*. Sorted by percentage of grandparents with primary/secondary education providing any care in 2019–20. Asterisks: ‘new’ (Wave 8, 2019–20) countries.* *Numerical values are available in the Supplementary Materials*

Figure 3 displays the predicted probabilities of providing any grandparental childcare by country, wave, and *education* (see Additional file 1: ﻿Table S3 for results referring to regular childcare by educational level). The ranking of countries in terms of grandparental childcare provided by less educated grandparents is similar to the general pattern shown in Fig. 1. Estimates for the group of more educated grandparents are less clear due to smaller sample sizes and, consequently, wider confidence intervals. Nonetheless, the propensity to provide grandchild care appears to be stable over time in both groups; however, there are a few noteworthy exceptions: Switzerland, for example, shows an increase in grandparental childcare provision, especially among *more* highly educated grandparents (of 19 pp and of 8 pp among less educated individuals), whereas we observe a similar increase (of 14 pp) among *less* highly educated grandparents in the Czech Republic (while the prevalence among more educated grandparents increases by 9 pp). The increases seen in the case of Switzerland and the Czech Republic are confirmed but are less pronounced, also when looking at regular grandchild care.

## Discussion

Building on data derived from the Survey of Health, Ageing and Retirement in Europe, the present study set out to investigate whether and how European grandparents’ provision of childcare may have changed over a period of 15 years (from 2004–07 to 2019–20) and how ‘new’ countries that were not included in earlier studies fit into previously identified patterns of grandparental childcare. Three main findings emerged from our descriptive analysis.

*First*, the overall prevalence and intensity of grandparental childcare in Europe remained fairly stable over time; if anything, a slight increase was detected. In the 13 countries that contributed data in both 2004–07 and 2019–20, on average, somewhat less than half of all grandparents provided any childcare during the twelve months preceding the surveys, and approximately one quarter cared for a grandchild on a regular basis. This overall picture of continuity rather than change is consistent with other recent research based on single countries indicating a high degree of temporal stability in aggregate intergenerational solidarity (Steinbach et al. 2020), including grandparenting (Pasqualini et al. 2021). We also observe this temporal stability in the provision of grandchild care when looking at trends within countries by gender and across educational groups.

*Second,* variations in grandchild care provision are generally the strongest across welfare state regimes (also see Bordone et al. 2023), but within countries, there is also considerable variation along the gender and education lines. Whereas grandmothers and grandfathers in France barely differ in their level of provision of both any and regular grandchild care (also see Craig et al. 2019), gender differences in, for example, Latvian grandparents’ engagement in childcare are substantial (with grandmothers being two to three times more likely to provide care than grandfathers). While similar levels of grandchild care provision are found across levels of education in the majority of countries, education seems to interact with the particular context of each country, indicating that in some cases (such as in Austria, Belgium, Denmark, Finland and Switzerland), more highly educated grandparents tend to be more likely to provide childcare, whereas the reverse is true elsewhere (such as in Hungary and Romania). This finding might reflect that both the greater *resources* available to support grandchildren in high-SES families (Zanasi and Sieben 2022) and the greater *demand* for grandparental childcare in low-SES families (Di Gessa et al. 2021) might matter—but to a different extent in different societal or institutional settings.

*Third*, resources and needs at the microlevel of the grandparent-(grand-)child dyad thus seem to interact with the generosity of welfare state services, women’s participation in the labor market, and cultural norms at the macrolevel. Previous studies have identified clusters of European countries along a continuum ranging from ‘familialism by default’ to ‘defamilialisation’, with distinct patterns of grandparental childcare provision (e.g., Bordone et al. 2017). Whereas this typology might still be applied to the ‘new’ countries covered in our analysis (with the addition of countries from central and southeastern Europe, for example, which correspond fairly well to the ‘familialism by default’ model), the ‘narrative’ of a negative macrolevel relationship between extensive and intensive grandparental childcare (e.g., Hank and Buber 2009) does not display as consistently anymore as it previously did. Nordic countries (Sweden and Denmark) as well as France—characterized by defamilialisation/supported familialism—are at the top of the distribution regarding the prevalence of grandparental childcare and the newly added country of Finland scores very similar to Sweden. Similarly, grandparents in Mediterranean countries and Poland exhibit a lower prevalence of grandparents providing childcare, but they are no longer at the bottom of the distribution because ‘new’ countries, such as Bulgaria, Latvia, and Slovakia, show even lower scores on the matter. Additionally, in this case, the addition of new countries in the study leaves the ranking largely consistent with the categorization established by Bordone et al. (2017), but we do not identify clear-cut geographical groups, especially for continental Europe: while Belgium and the Netherlands are at the very top of the distribution in terms of the prevalence of grandchild care, Luxembourg, Germany, Austria and the Czech Republic occupy a middle position; also, grandparents in Estonia and Hungary show a lower likelihood of providing childcare than their counterparts in the Mediterranean countries.

In the case of regular (that is, at least weekly) childcare, the picture becomes even more complicated. If we only considered northern and Mediterranean countries, we could conclude that a north‒south gradient persists between prevalence and intensity; however, updated statistics that include the ‘new’ countries provide a more scattered picture: countries such as Belgium and the Netherlands show a very high prevalence of both any and weekly care, while in the new eastern European countries, that prevalence remains at the bottom of the ranking, with a very low prevalence for both any and weekly care. The complexity of the interaction between country- and individual-level factors—in an overlapping of norms and preferences as well as needs and opportunities—thus appears to have increased. Specifically, more work is needed to improve our understanding of central and southeastern European countries’ family (policy) regimes.

This study is not without limitations. Ideally, we would have used data from repeated cross-sectional surveys to compare the prevalence of grandchild care over time. However, cross-national data of this sort are not available. Like all studies based on longitudinal samples, our estimates might be affected by attrition. To address this issue, at least partially, we employed calibrated weights provided by SHARE. Repeated cross-sectional data would also have likely resulted in more similar compositions of samples across time (for example, in terms of age distributions). As we noted above, this was not the case in our data, where individuals in Wave 8 were on average older than at baseline. However, robustness checks confirmed the stability of the results (see Additional file 1: Figure S1).

We provided an up-to-date cross-national overview of grandchild care prevalence across European countries, as well as an analysis of its evolution over a 15-year period. Our analysis revealed a high degree of stability in grandparents’ provision of childcare, which tended to increase rather than decline. The crucial role grandparents play as care providers for children is thus confirmed in all European countries, although the intensity of this activity varies depending on the context. Our descriptive study establishes the groundwork for theoretical and empirical advancement in grandparenthood research. In particular, an important avenue for future research is to examine the micro-, meso-, and macrolevel determinants of both stability *and* changes in childcare provision. Further improving our understanding of these factors seems desirable, because grandparents’ (potential) involvement in childcare is not only relevant for intergenerational family functioning, but also has important demographic implications, specifically with regard to fertility decisions (e.g., Rutigliano 2020; 2023).

### Supplementary Information

Below is the link to the electronic supplementary material.

**Additional file 1**.
