# Supplementary material for: The prevalence of grandparental childcare in Europe: a research update
Source: Eur J Ageing. 2023 Sep 25;20(1):37. doi: 10.1007/s10433-023-00785-8 (PMC10519902; doi:10.1007/s10433-023-00785-8)
Supplement: Supplementary file 1 — Additional file 1. [file 10433_2023_785_MOESM1_ESM.docx]

**The prevalence of grandparental childcare in Europe:**

**A research update**

***Online Supplementary Material***

### Figure S1. Sample distribution by age at baseline (2004-07) and in Wave 8 (2019-20), without (left panel) and with (right panel) application of calibrated weights


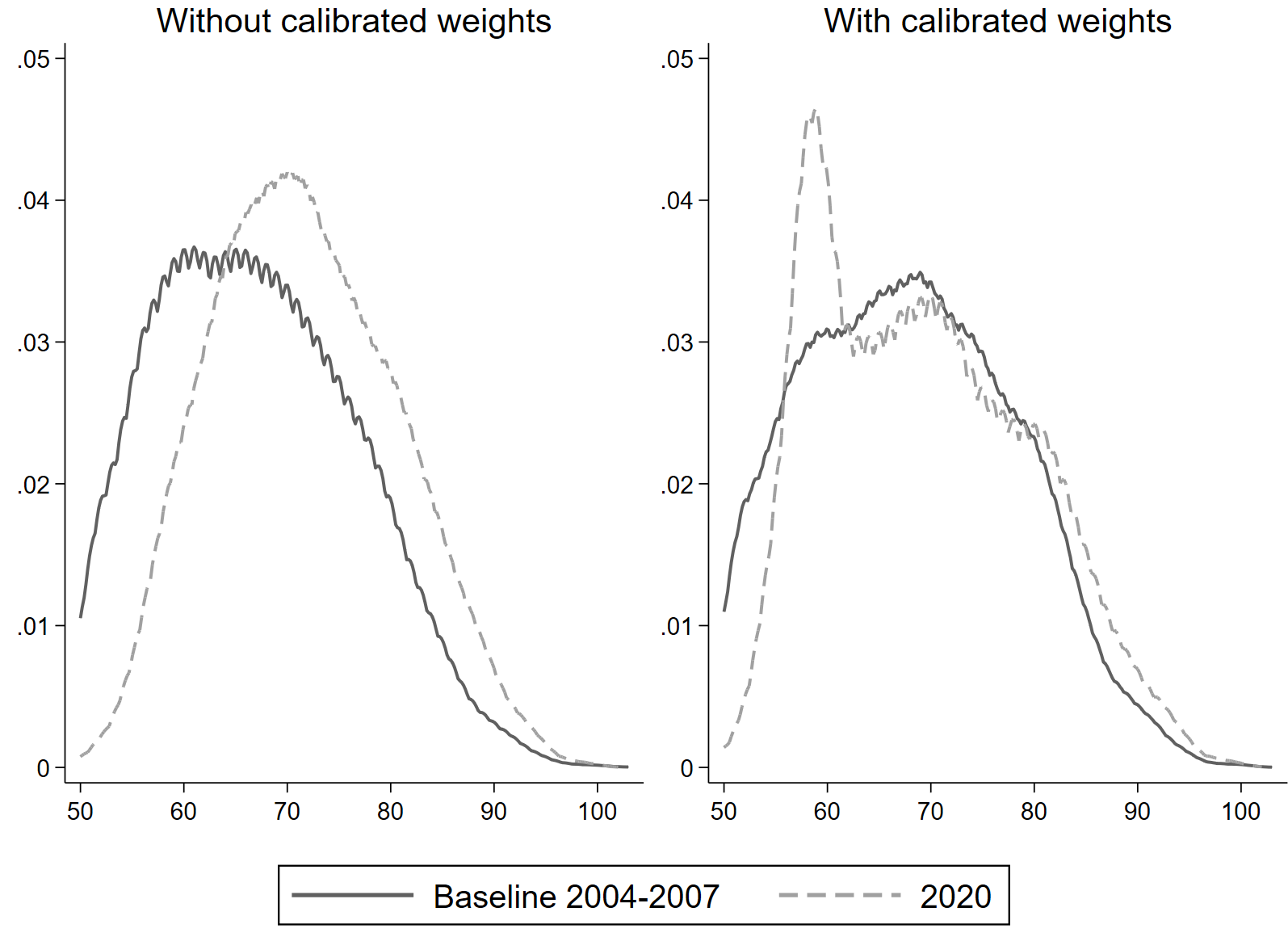


### Table S1. Predicted probabilities from age-adjusted logistic regression models, between baseline (2004-07), Wave 6 (2015), and Wave 8 (2019-20), referring to Figure 1. Calibrated weights applied.

|  | Any Care | | | | | | | | | Weekly Care | | | | | | | | |
| --- | --- | --- | --- | --- | --- | --- | --- | --- | --- | --- | --- | --- | --- | --- | --- | --- | --- | --- |
|  | Baseline  (2004-2007) | | Wave 6  (2015) | | Wave 8  (2020) | | Difference  Wave8-baseline | | | Baseline  (2004-2007) | | Wave 6  (2015) | | Wave 8  (2020) | | Difference  Wave8-baseline | | |
|  | *Prob.* | *SE* | *Prob.* | *SE* | *Prob.* | *SE* | *Prob.* | | *SE* | *Prob.* | *SE* | *Prob.* | *SE* | *Prob.* | *SE* | *Prob.* | | *SE* |
| Austria | .39 | .01 | .40 | .01 | .45 | .03 | .06 | * | .03 | .21 | .01 | .25 | .01 | .27 | .03 | .05 |  | .03 |
| Germany | .37 | .01 | .41 | .01 | .46 | .01 | .09 | *** | .02 | .20 | .01 | .22 | .01 | .23 | .01 | .03 |  | .02 |
| Sweden | .45 | .01 | .53 | .01 | .55 | .02 | .09 | *** | .02 | .14 | .01 | .14 | .01 | .14 | .01 | .00 |  | .02 |
| Netherlands | .52 | .01 | n.a. | n.a. | .60 | .01 | .08 | *** | .02 | .24 | .01 | . | . | .34 | .02 | .10 | *** | .02 |
| Spain | .39 | .01 | .39 | .02 | .43 | .05 | .04 |  | .05 | .26 | .01 | .28 | .02 | .25 | .04 | -.01 |  | .04 |
| Italy | .41 | .01 | .38 | .01 | .37 | .03 | -.04 |  | .03 | .32 | .01 | .30 | .01 | .31 | .03 | -.01 |  | .03 |
| France | .46 | .01 | .49 | .01 | .58 | .01 | .12 | *** | .02 | .19 | .01 | .18 | .01 | .24 | .01 | .05 | ** | .02 |
| Denmark | .50 | .01 | .50 | .01 | .56 | .01 | .06 | *** | .02 | .14 | .01 | .15 | .01 | .17 | .01 | .03 | * | .01 |
| Greece | .48 | .01 | .46 | .01 | .38 | .01 | -.11 | *** | .02 | .35 | .01 | .30 | .01 | .25 | .01 | -.11 | *** | .02 |
| Switzerland | .42 | .02 | .47 | .02 | .52 | .03 | .10 | ** | .03 | .24 | .02 | .28 | .02 | .25 | .02 | .01 |  | .03 |
| Belgium | .51 | .01 | .52 | .01 | .60 | .02 | .09 | *** | .02 | .30 | .01 | .30 | .01 | .39 | .02 | .09 | *** | .02 |
| Czech Republic | .33 | .01 | .43 | .01 | .47 | .02 | .14 | *** | .03 | .16 | .01 | .25 | .01 | .26 | .02 | .10 | *** | .03 |
| Poland | .39 | .01 | .33 | .01 | .38 | .01 | .00 |  | .02 | .27 | .01 | .21 | .01 | .23 | .01 | -.04 | * | .02 |
| Luxembourg | n.a. | n.a. | .47 | .02 | .56 | .02 | n.a. | n.a. | n.a. | n.a. | n.a. | .31 | .02 | .36 | .03 | n.a. | n.a. | n.a. |
| Hungary | n.a. | n.a. | n.a. | n.a. | .39 | .05 | n.a. | n.a. | n.a. | n.a. | n.a. | n.a. | n.a. | .20 | .04 | n.a. | n.a. | n.a. |
| Slovenia | n.a. | n.a. | .41 | .01 | .46 | .01 | n.a. | n.a. | n.a. | n.a. | n.a. | .24 | .01 | .28 | .01 | n.a. | n.a. | n.a. |
| Estonia | n.a. | n.a. | .37 | .01 | .38 | .01 | n.a. | n.a. | n.a. | n.a. | n.a. | .14 | .01 | .14 | .01 | n.a. | n.a. | n.a. |
| Croatia | n.a. | n.a. | .40 | .01 | .39 | .02 | n.a. | n.a. | n.a. | n.a. | n.a. | .26 | .01 | .25 | .02 | n.a. | n.a. | n.a. |
| Lithuania | n.a. | n.a. | n.a. | n.a. | .26 | .01 | n.a. | n.a. | n.a. | n.a. | n.a. | n.a. | n.a. | .13 | .01 | n.a. | n.a. | n.a. |
| Bulgaria | n.a. | n.a. | n.a. | n.a. | .26 | .02 | n.a. | n.a. | n.a. | n.a. | n.a. | n.a. | n.a. | .18 | .02 | n.a. | n.a. | n.a. |
| Cyprus | n.a. | n.a. | n.a. | n.a. | .38 | .03 | n.a. | n.a. | n.a. | n.a. | n.a. | n.a. | n.a. | .35 | .03 | n.a. | n.a. | n.a. |
| Finland | n.a. | n.a. | n.a. | n.a. | .52 | .03 | n.a. | n.a. | n.a. | n.a. | n.a. | n.a. | n.a. | .15 | .02 | n.a. | n.a. | n.a. |
| Latvia | n.a. | n.a. | n.a. | n.a. | .24 | .02 | n.a. | n.a. | n.a. | n.a. | n.a. | n.a. | n.a. | .12 | .01 | n.a. | n.a. | n.a. |
| Malta | n.a. | n.a. | n.a. | n.a. | .45 | .02 | n.a. | n.a. | n.a. | n.a. | n.a. | n.a. | n.a. | .35 | .02 | n.a. | n.a. | n.a. |
| Romania | n.a. | n.a. | n.a. | n.a. | .26 | .02 | n.a. | n.a. | n.a. | n.a. | n.a. | n.a. | n.a. | .19 | .02 | n.a. | n.a. | n.a. |
| Slovakia | n.a. | n.a. | n.a. | n.a. | .28 | .02 | n.a. | n.a. | n.a. | n.a. | n.a. | n.a. | n.a. | .13 | .01 | n.a. | n.a. | n.a. |
| Mean | .42 | .00 | .42 | .00 | .46 | .01 | .04 | *** | .01 | .24 | .00 | .23 | .00 | .25 | .01 | .01 |  | .01 |

*Note:* *p< .05, **p< .01, ***p< .001

### Table S2. Predicted probabilities from age-adjusted logistic regression models, between baseline (2004-07), Wave 6 (2015), and Wave 8 (2019-20), by gender, referring to Figure 2. Calibrated weights applied.

|  | Any Care | | | | | | | | Weekly Care | | | | | | | |
| --- | --- | --- | --- | --- | --- | --- | --- | --- | --- | --- | --- | --- | --- | --- | --- | --- |
|  | Men | | | | Women | | | | Men | | | | Women | | | |
|  | Baseline  (2004-2007) | | Wave 8  (2020) | | Baseline  (2004-2007) | | Wave 8  (2020) | | Baseline  (2004-2007) | | Wave 8  (2020) | | Baseline  (2004-2007) | | Wave 8  (2020) | |
|  | *Prob.* | *SE* | *Prob.* | *SE* | *Prob.* | *SE* | *Prob.* | *SE* | *Prob.* | *SE* | *Prob.* | *SE* | *Prob.* | *SE* | *Prob.* | *SE* |
| Austria | .41 | .02 | .46 | .04 | .38 | .02 | .44 | .03 | .20 | .02 | .24 | .04 | .22 | .02 | .29 | .03 |
| Germany | .37 | .02 | .44 | .02 | .38 | .01 | .47 | .02 | .18 | .01 | .18 | .02 | .22 | .01 | .26 | .02 |
| Sweden | .42 | .02 | .51 | .03 | .49 | .01 | .58 | .02 | .12 | .01 | .13 | .02 | .15 | .01 | .15 | .02 |
| Netherlands | .53 | .02 | .57 | .02 | .52 | .01 | .62 | .01 | .23 | .01 | .31 | .03 | .25 | .01 | .36 | .03 |
| Spain | .35 | .02 | .40 | .08 | .41 | .02 | .46 | .04 | .24 | .02 | .22 | .06 | .27 | .02 | .29 | .04 |
| Italy | .34 | .02 | .34 | .04 | .45 | .02 | .38 | .03 | .26 | .02 | .26 | .03 | .35 | .01 | .34 | .03 |
| France | .44 | .02 | .58 | .02 | .48 | .01 | .58 | .02 | .16 | .01 | .22 | .02 | .21 | .01 | .25 | .02 |
| Denmark | .48 | .02 | .55 | .02 | .52 | .01 | .57 | .02 | .12 | .01 | .15 | .02 | .16 | .01 | .19 | .02 |
| Greece | .47 | .02 | .32 | .03 | .48 | .01 | .41 | .02 | .35 | .02 | .20 | .02 | .35 | .01 | .28 | .02 |
| Switzerland | .39 | .03 | .46 | .05 | .45 | .02 | .58 | .02 | .18 | .02 | .17 | .02 | .28 | .02 | .33 | .03 |
| Belgium | .50 | .02 | .57 | .03 | .52 | .01 | .62 | .02 | .29 | .01 | .38 | .03 | .30 | .01 | .40 | .03 |
| Czech Republic | .30 | .02 | .43 | .04 | .37 | .02 | .51 | .02 | .15 | .02 | .21 | .04 | .18 | .01 | .31 | .03 |
| Poland | .36 | .02 | .35 | .02 | .41 | .01 | .41 | .02 | .24 | .02 | .20 | .02 | .29 | .01 | .25 | .02 |
| Luxembourg | n.a. | n.a. | .52 | .04 | n.a. | n.a. | .58 | .03 | n.a. | n.a. | .31 | .04 | n.a. | n.a. | .39 | .04 |
| Hungary | n.a. | n.a. | .34 | .06 | n.a. | n.a. | .43 | .06 | n.a. | n.a. | .21 | .07 | n.a. | n.a. | .20 | .05 |
| Slovenia | n.a. | n.a. | .46 | .02 | n.a. | n.a. | .46 | .01 | n.a. | n.a. | .27 | .03 | n.a. | n.a. | .28 | .02 |
| Estonia | n.a. | n.a. | .32 | .02 | n.a. | n.a. | .43 | .02 | n.a. | n.a. | .12 | .01 | n.a. | n.a. | .16 | .01 |
| Croatia | n.a. | n.a. | .36 | .03 | n.a. | n.a. | .41 | .02 | n.a. | n.a. | .22 | .03 | n.a. | n.a. | .27 | .02 |
| Lithuania | n.a. | n.a. | .20 | .02 | n.a. | n.a. | .31 | .02 | n.a. | n.a. | .13 | .02 | n.a. | n.a. | .13 | .01 |
| Bulgaria | n.a. | n.a. | .24 | .03 | n.a. | n.a. | .27 | .02 | n.a. | n.a. | .15 | .03 | n.a. | n.a. | .19 | .02 |
| Cyprus | n.a. | n.a. | .33 | .05 | n.a. | n.a. | .43 | .04 | n.a. | n.a. | .31 | .05 | n.a. | n.a. | .38 | .04 |
| Finland | n.a. | n.a. | .52 | .05 | n.a. | n.a. | .53 | .03 | n.a. | n.a. | .15 | .03 | n.a. | n.a. | .16 | .03 |
| Latvia | n.a. | n.a. | .16 | .03 | n.a. | n.a. | .29 | .03 | n.a. | n.a. | .06 | .02 | n.a. | n.a. | .17 | .02 |
| Malta | n.a. | n.a. | .43 | .03 | n.a. | n.a. | .47 | .03 | n.a. | n.a. | .33 | .03 | n.a. | n.a. | .37 | .03 |
| Romania | n.a. | n.a. | .23 | .03 | n.a. | n.a. | .28 | .02 | n.a. | n.a. | .16 | .02 | n.a. | n.a. | .22 | .02 |
| Slovakia | n.a. | n.a. | .24 | .02 | n.a. | n.a. | .32 | .02 | n.a. | n.a. | .11 | .02 | n.a. | n.a. | .14 | .02 |
| Mean | .39 | .01 | .43 | .01 | .43 | .01 | .47 | .01 | .21 | .01 | .21 | .01 | .26 | .00 | .27 | .01 |

*Note:* *p< .05, **p< .01, ***p< .001

### Table S3. Predicted probabilities from age-adjusted logistic regression models, between baseline (2004-07), Wave 6 (2015), and Wave 8 (2019-20), by education, referring to Figure 3. Calibrated weights applied.

|  | Any Care | | | | | | | | Weekly Care | | | | | | | |
| --- | --- | --- | --- | --- | --- | --- | --- | --- | --- | --- | --- | --- | --- | --- | --- | --- |
|  | Primary/Secondary | | | | Tertiary | | | | Primary/Secondary | | | | Tertiary | | | |
|  | Baseline  (2004-2007) | | Wave 8 (2020) | | Baseline  (2004-2007) | | Wave 8  (2020) | | Baseline  (2004-2007) | | Wave 8  (2020) | | Baseline  (2004-2007) | | Wave 8  (2020) | |
|  | *Prob.* | *SE* | *Prob.* | *SE* | *Prob.* | *SE* | *Prob.* | *SE* | *Prob.* | *SE* | *Prob.* | *SE* | *Prob.* | *SE* | *Prob.* | *SE* |
| Austria | .38 | .02 | .42 | .03 | .48 | .04 | .56 | .04 | .21 | .01 | .26 | .03 | .23 | .03 | .27 | .04 |
| Germany | .36 | .01 | .44 | .02 | .43 | .02 | .50 | .02 | .20 | .01 | .23 | .01 | .20 | .02 | .21 | .03 |
| Sweden | .45 | .01 | .54 | .03 | .50 | .03 | .59 | .03 | .14 | .01 | .14 | .02 | .16 | .02 | .15 | .02 |
| Netherlands | .52 | .01 | .58 | .02 | .58 | .03 | .64 | .02 | .25 | .01 | .35 | .02 | .20 | .02 | .33 | .03 |
| Spain | .39 | .01 | .43 | .05 | .40 | .06 | .44 | .07 | .26 | .01 | .25 | .04 | .27 | .05 | .30 | .07 |
| Italy | .40 | .01 | .38 | .03 | .55 | .08 | .42 | .07 | .31 | .01 | .31 | .02 | .39 | .08 | .35 | .06 |
| France | .45 | .01 | .56 | .02 | .56 | .03 | .64 | .03 | .19 | .01 | .26 | .02 | .19 | .02 | .20 | .02 |
| Denmark | .48 | .01 | .51 | .02 | .56 | .02 | .63 | .02 | .14 | .01 | .15 | .01 | .15 | .02 | .21 | .02 |
| Greece | .48 | .01 | .37 | .02 | .52 | .07 | .41 | .04 | .35 | .01 | .24 | .02 | .36 | .07 | .29 | .03 |
| Switzerland | .42 | .02 | .50 | .03 | .42 | .07 | .63 | .05 | .24 | .02 | .25 | .03 | .19 | .06 | .29 | .04 |
| Belgium | .48 | .01 | .54 | .02 | .63 | .02 | .70 | .02 | .29 | .01 | .34 | .02 | .33 | .02 | .48 | .03 |
| Czech Republic | .32 | .01 | .46 | .03 | .45 | .04 | .54 | .06 | .15 | .01 | .25 | .03 | .28 | .05 | .30 | .05 |
| Poland | .38 | .01 | .37 | .01 | .51 | .06 | .52 | .04 | .27 | .01 | .23 | .01 | .31 | .05 | .28 | .04 |
| Luxembourg | n.a. | n.a. | .53 | .03 | n.a. | n.a. | .76 | .05 | n.a. | n.a. | .35 | .03 | n.a. | n.a. | .44 | .06 |
| Hungary | n.a. | n.a. | .41 | .05 | n.a. | n.a. | .32 | .11 | n.a. | n.a. | .23 | .04 | n.a. | n.a. | .13 | .06 |
| Slovenia | n.a. | n.a. | .44 | .01 | n.a. | n.a. | .54 | .03 | n.a. | n.a. | .26 | .02 | n.a. | n.a. | .36 | .03 |
| Estonia | n.a. | n.a. | .36 | .01 | n.a. | n.a. | .45 | .02 | n.a. | n.a. | .14 | .01 | n.a. | n.a. | .16 | .02 |
| Croatia | n.a. | n.a. | .39 | .02 | n.a. | n.a. | .37 | .05 | n.a. | n.a. | .25 | .02 | n.a. | n.a. | .23 | .04 |
| Lithuania | n.a. | n.a. | .24 | .02 | n.a. | n.a. | .31 | .02 | n.a. | n.a. | .13 | .01 | n.a. | n.a. | .13 | .02 |
| Bulgaria | n.a. | n.a. | .26 | .02 | n.a. | n.a. | .27 | .05 | n.a. | n.a. | .17 | .02 | n.a. | n.a. | .20 | .05 |
| Cyprus | n.a. | n.a. | .37 | .04 | n.a. | n.a. | .53 | .08 | n.a. | n.a. | .32 | .03 | n.a. | n.a. | .49 | .08 |
| Finland | n.a. | n.a. | .41 | .05 | n.a. | n.a. | .68 | .04 | n.a. | n.a. | .13 | .03 | n.a. | n.a. | .18 | .04 |
| Latvia | n.a. | n.a. | .24 | .02 | n.a. | n.a. | .23 | .04 | n.a. | n.a. | .12 | .02 | n.a. | n.a. | .15 | .03 |
| Malta | n.a. | n.a. | .46 | .02 | n.a. | n.a. | .44 | .12 | n.a. | n.a. | .36 | .02 | n.a. | n.a. | .33 | .10 |
| Romania | n.a. | n.a. | .26 | .02 | n.a. | n.a. | .19 | .07 | n.a. | n.a. | .20 | .02 | n.a. | n.a. | .08 | .04 |
| Slovakia | n.a. | n.a. | .29 | .02 | n.a. | n.a. | .30 | .10 | n.a. | n.a. | .13 | .01 | n.a. | n.a. | .08 | .04 |
| Mean | .41 | .00 | .43 | .01 | .49 | .01 | .54 | .01 | .24 | .00 | .25 | .01 | .22 | .01 | .23 | .01 |

*Note:* *p< .05, **p< .01, ***p< .001

### Table S4. Comparison of predicted probabilities from different model specifications (unadjusted and only on respondents aged 60+) between baseline (2004-07), and Wave 8 (2019-20)

|  | Unadjusted | | | | | | | | 60+ only | | | | | | | |
| --- | --- | --- | --- | --- | --- | --- | --- | --- | --- | --- | --- | --- | --- | --- | --- | --- |
|  | Any | | | | Weekly | | | | Any | | | | Weekly | | | |
|  | Baseline  (2004-2007) | | Wave 8  (2020) | | Baseline  (2004-2007) | | Wave 8  (2020) | | Baseline  (2004-2007) | | Wave 8  (2020) | | Baseline  (2004-2007) | | Wave 8  (2020) | |
|  | *Prob.* | *SE* | *Prob.* | *SE* | *Prob.* | *SE* | *Prob.* | *SE* | *Prob.* | *SE* | *Prob.* | *SE* | *Prob.* | *SE* | *Prob.* | *SE* |
| Austria | .42 | .02 | .47 | .03 | .21 | .01 | .27 | .03 | .33 | .02 | .40 | .02 | .17 | .01 | .25 | .02 |
| Germany | .38 | .01 | .45 | .02 | .20 | .01 | .23 | .01 | .32 | .01 | .39 | .01 | .18 | .01 | .20 | .01 |
| Sweden | .47 | .01 | .53 | .02 | .14 | .01 | .14 | .01 | .41 | .01 | .53 | .01 | .13 | .01 | .15 | .01 |
| Netherlands | .56 | .01 | .54 | .02 | .24 | .01 | .34 | .02 | .46 | .01 | .51 | .01 | .21 | .01 | .28 | .01 |
| Spain | .37 | .01 | .40 | .04 | .26 | .01 | .25 | .04 | .35 | .01 | .38 | .03 | .23 | .01 | .26 | .02 |
| Italy | .40 | .01 | .34 | .02 | .32 | .01 | .31 | .03 | .37 | .01 | .36 | .02 | .28 | .01 | .29 | .02 |
| France | .48 | .01 | .57 | .02 | .19 | .01 | .24 | .01 | .41 | .01 | .53 | .01 | .17 | .01 | .21 | .01 |
| Denmark | .56 | .01 | .56 | .01 | .14 | .01 | .17 | .01 | .44 | .01 | .51 | .01 | .13 | .01 | .17 | .01 |
| Greece | .45 | .01 | .32 | .01 | .35 | .01 | .25 | .01 | .41 | .01 | .31 | .01 | .30 | .01 | .20 | .01 |
| Switzerland | .42 | .02 | .49 | .03 | .24 | .02 | .25 | .02 | .37 | .02 | .49 | .01 | .21 | .02 | .27 | .02 |
| Belgium | .53 | .01 | .60 | .02 | .30 | .01 | .39 | .02 | .45 | .01 | .53 | .01 | .26 | .01 | .33 | .02 |
| Czech Republic | .40 | .02 | .50 | .03 | .16 | .01 | .26 | .02 | .26 | .01 | .40 | .01 | .13 | .01 | .21 | .01 |
| Poland | .45 | .01 | .41 | .01 | .27 | .01 | .23 | .01 | .32 | .01 | .31 | .01 | .22 | .01 | .19 | .01 |
| Luxembourg | n.a. | n.a. | .54 | .03 | n.a. | n.a. | .36 | .03 | n.a. | n.a. | .50 | .02 | n.a. | n.a. | .33 | .02 |
| Hungary | n.a. | n.a. | .37 | .04 | n.a. | n.a. | .20 | .04 | n.a. | n.a. | .31 | .04 | n.a. | n.a. | .11 | .02 |
| Slovenia | n.a. | n.a. | .47 | .02 | n.a. | n.a. | .28 | .01 | n.a. | n.a. | .36 | .01 | n.a. | n.a. | .22 | .01 |
| Estonia | n.a. | n.a. | .39 | .01 | n.a. | n.a. | .14 | .01 | n.a. | n.a. | .33 | .01 | n.a. | n.a. | .13 | .01 |
| Croatia | n.a. | n.a. | .40 | .02 | n.a. | n.a. | .25 | .02 | n.a. | n.a. | .33 | .02 | n.a. | n.a. | .20 | .01 |
| Lithuania | n.a. | n.a. | .28 | .02 | n.a. | n.a. | .13 | .01 | n.a. | n.a. | .21 | .01 | n.a. | n.a. | .11 | .01 |
| Bulgaria | n.a. | n.a. | .27 | .02 | n.a. | n.a. | .18 | .02 | n.a. | n.a. | .21 | .02 | n.a. | n.a. | .13 | .01 |
| Cyprus | n.a. | n.a. | .41 | .04 | n.a. | n.a. | .35 | .03 | n.a. | n.a. | .36 | .03 | n.a. | n.a. | .33 | .03 |
| Finland | n.a. | n.a. | .51 | .03 | n.a. | n.a. | .15 | .02 | n.a. | n.a. | .46 | .03 | n.a. | n.a. | .14 | .02 |
| Latvia | n.a. | n.a. | .25 | .02 | n.a. | n.a. | .12 | .01 | n.a. | n.a. | .21 | .02 | n.a. | n.a. | .12 | .01 |
| Malta | n.a. | n.a. | .45 | .02 | n.a. | n.a. | .35 | .02 | n.a. | n.a. | .40 | .02 | n.a. | n.a. | .32 | .02 |
| Romania | n.a. | n.a. | .27 | .02 | n.a. | n.a. | .19 | .02 | n.a. | n.a. | .22 | .02 | n.a. | n.a. | .17 | .02 |
| Slovakia | n.a. | n.a. | .31 | .02 | n.a. | n.a. | .13 | .01 | n.a. | n.a. | .22 | .02 | n.a. | n.a. | .09 | .01 |
| Mean | .43 | .00 | .45 | .01 | .24 | .00 | .25 | .01 | .36 | .00 | .40 | .01 | .21 | .00 | .22 | .00 |

*Note:* *p< .05, **p< .01, ***p< .001
